# Coherent Multimodal Sensory Information Allows Switching between Gravitoinertial Contexts

**DOI:** 10.3389/fphys.2017.00290

**Published:** 2017-05-11

**Authors:** Marie Barbiero, Célia Rousseau, Charalambos Papaxanthis, Olivier White

**Affiliations:** ^1^Université de Bourgogne Franche-Comté, Cognition Action et Plasticité Sensorimotrice UMR1093Dijon, France; ^2^Institut National de Santé et de Recherche Médicale, Cognition Action et Plasticité Sensorimotrice UMR1093Dijon, France

**Keywords:** multisensory information, feedback, switching, grip force, human centrifuge, gravity, internal model

## Abstract

Whether the central nervous system is capable to switch between contexts critically depends on experimental details. Motor control studies regularly adopt robotic devices to perturb the dynamics of a certain task. Other approaches investigate motor control by altering the gravitoinertial context itself as in parabolic flights and human centrifuges. In contrast to conventional robotic experiments, where only the hand is perturbed, these gravitoinertial or immersive settings coherently plunge participants into new environments. However, radically different they are, perfect adaptation of motor responses are commonly reported. In object manipulation tasks, this translates into a good matching of the grasping force or grip force to the destabilizing load force. One possible bias in these protocols is the predictability of the forthcoming dynamics. Here we test whether the successful switching and adaptation processes observed in immersive environments are a consequence of the fact that participants can predict the perturbation schedule. We used a short arm human centrifuge to decouple the effects of space and time on the dynamics of an object manipulation task by adding an unnatural explicit position-dependent force. We created different dynamical contexts by asking 20 participants to move the object at three different paces. These contextual sessions were interleaved such that we could simulate concurrent learning. We assessed adaptation by measuring how grip force was adjusted to this unnatural load force. We found that the motor system can switch between new unusual dynamical contexts, as reported by surprisingly well-adjusted grip forces, and that this capacity is not a mere consequence of the ability to predict the time course of the upcoming dynamics. We posit that a coherent flow of multimodal sensory information born in a homogeneous milieu allows switching between dynamical contexts.

## Introduction

Consider a worker whose job is to sort Christmas packages of varying size and weight into bins, bags, or slots. Each of these packages will have different inertial properties and will impose different loads on the arm. The physical properties of these objects are not fixed but vary according to a given statistical distribution that depends both on object properties and on the sequence of planned movements. Despite the fact that variability occurs on a movement basis, this context is predictable in the sense that the worker can estimate the upcoming mechanical properties based on visual cues. If the worker carries out this task for a prolonged time, s/he will adjust her/his motor plan according to the object and action. In other words, motor adaptation and context switching will occur (Kawato, 1999).

Studies regularly use robotic devices to perturb the dynamics of motor tasks. This allows testing of how specific parameters such as stiffness (Descoins et al., 2006), viscosity (Shadmehr and Mussa-Ivaldi, 1994) and inertia (Wang and Sainburg, 2004) are taken into account by the central nervous system to plan efficient actions. Robot-based investigations highlighted limitations of the brain to concurrently learn different task dynamics (Gandolfo et al., 1996; Conditt et al., 1997; Karniel and Mussa-Ivaldi, 2002), even when the expected dynamics are made fully predictable through the use of explicit cues, such as the association of a color to a direction of a forthcoming perturbation (Krakauer et al., 1999; Osu et al., 2004). In other contexts, however, the motor system is quite capable of learning different dynamics. If one moves the arm alone or the arm linked to an unfamiliar object, two parallel predictive strategies are formed by the brain (Kluzik et al., 2008). The same observation has been reported with different objects and one or two hands (Ahmed et al., 2008; White and Diedrichsen, 2008). Furthermore, this efficient concurrent learning is also possible if control policies—or predictive strategies—are associated to different contexts, such as a leftward or rightward perturbing force field (White and Diedrichsen, 2013). Whether participants can or cannot switch between contexts critically depends on experimental details.

Motor adaptation has also been probed using other approaches. For instance, parabolic flights and human centrifuges provide unique means to alter the whole gravitational or gravitoinertial environment. In the former, the participant is immersed into a repeated gravitational profile (e.g., 1, 1.8, 0, 1.8 g and back to 1 g, where 1 g is Earth gravity). Human centrifuges allow programming an arbitrary gravitoinertial environment (e.g., staircase function from 1 to 3 g). In contrast to conventional robotic experiments, where only the hand is perturbed, parabolic flight and rotating-room environments plunge the subject into a radically new setting. Nearly perfect adaptation of motor responses in those challenging environments were observed in dexterous manipulation (Augurelle et al., 2003; White et al., 2005; Göbel et al., 2006; Mierau et al., 2008; Crevecoeur et al., 2009b), arm movements (Papaxanthis et al., 1998; White et al., 2008) and more realistic tasks (Steinberg et al., 2015).

In previous investigations involving movements in altered gravitoinertial environment, the dynamic consequences of actions only depended on time. In other words, external constraints were constant in the Euclidian space, but could vary according to a predefined experimental schedule and/or self-generated movements. A question arises as to whether adaptation observed in the above studies is a mere consequence of the fact gravitoinertial profiles vary over time and can be predicted? A structural decoupling between underlying variables—space and time—may highlight different time scales of adaptation. Here, we test the ability of participants to adapt and switch between very unusual dynamical contexts generated by rotation of a short-arm human centrifuge. Space and time are decoupled because the gravitoinertial vector can vary significantly along a short movement amplitude. In other words, this also means that local gravity will be different according to where the object is in space, independently of time (see Methods). Twenty participants cyclically moved an object along the head-to-foot body axis, aligned with the gravitoinertial vector, induced by rotation of the centrifuge. We measured adaptation through the robust paradigm of grip force adjustments to load force (Westling and Johansson, 1984; Jaric et al., 2005). When moving an object with a precision grip configuration (thumb opposing the index finger), the brain must estimate the dynamical consequences of the movement on the tangential destabilizing force (load force). This is necessary to estimate the required grasping force (grip force) and avoid accidental slips. Many studies have shown very good adjustments of grip force to a variety of physical object parameters (mass, texture, shape, friction) or environments (force fields, gravitational fields). We created different dynamics by instructing participants to perform the movements at three different paces. These contextual sessions were interleaved such that we could simulate concurrent learning at two time scales (within a session and between sessions). We speculate that failure to adapt grip force like in other similar experiments would underline the fact that time alone is not sufficient to predict the forthcoming dynamics. In contrast, successful grip force adaptation would demonstrate the flexibility of the nervous system to efficiently switch between these truly novel dynamics.

## Materials and methods

### Participants and ethical considerations

Twenty healthy, un-trained, non-obese, non-smoking men (*n* = 10, 29.5 ± 5.3 years old, 178.9 ± 4.6 cm, BMI 25.1 ± 2.0 kg/m^2^) and women (*n* = 10, 27.6 ± 4.6 years old, 165.1 ± 4.8 cm, BMI 21.9 ± 1.9 kg/m^2^) without histories of vasovagal syncope or cardiovascular problems took part in this protocol. Each participant received a comprehensive medical examination by a medical doctor from MEDES (French Institute for Space Medicine and Physiology) prior to participation. Inclusion criterial were: age between 20 and 40 years old, BMI < 30 kg/m^2^, normal clinical examination, normal electrocardiogram and arterial pressure, signed consent and enrolled in the French social security system. Those already participating in another biomedical test, who did not comply with any of the above inclusion criteria or under medication for 8 days before the experiment were not retained for this experiment. The experiment could be interrupted at any time upon participants' request or his/her health status under constant monitoring by a medical supervisor. We had to interrupt the experiment during the last session (see Experimental procedures) for five female participants who showed signs of motion sickness. This did not impact our results since we designed the experiment in such a way to accumulate a large data set in a short amount of time. Consequently, we had only slightly less data (30% drop out) for these five participants.

The experiment took place at MEDES, Toulouse (France). None had previously experienced hypergravity in a short arm human centrifuge (SAHC) and the preparatory visit did not include a familiarization session in order to keep them naïve with respect to this new environment. The study was conducted in accordance with the ethical practices stipulated in the Declaration of Helsinki (1964). Ethics approval was obtained by MEDES (2014-A00212-45). All volunteers signed the informed consent form, which is stored at MEDES.

### Experimental procedures

The participant laid on a horizontal bed and was monitored with heart rate and arterial pressure systems using non-invasive photoplethysmography (Portapres: FMS, the Netherlands). The Portapres finger cuff was placed on the resting hand during the task. Her/his head rested on a thin pillow and her/his feet contacted a rigid metallic platform. The participant was then equipped with headphones in order to maintain contact with the operator in the control room. Visual feedback of the environment was prevented by placing an opaque ventilated box above the head.

**Figure 1:**
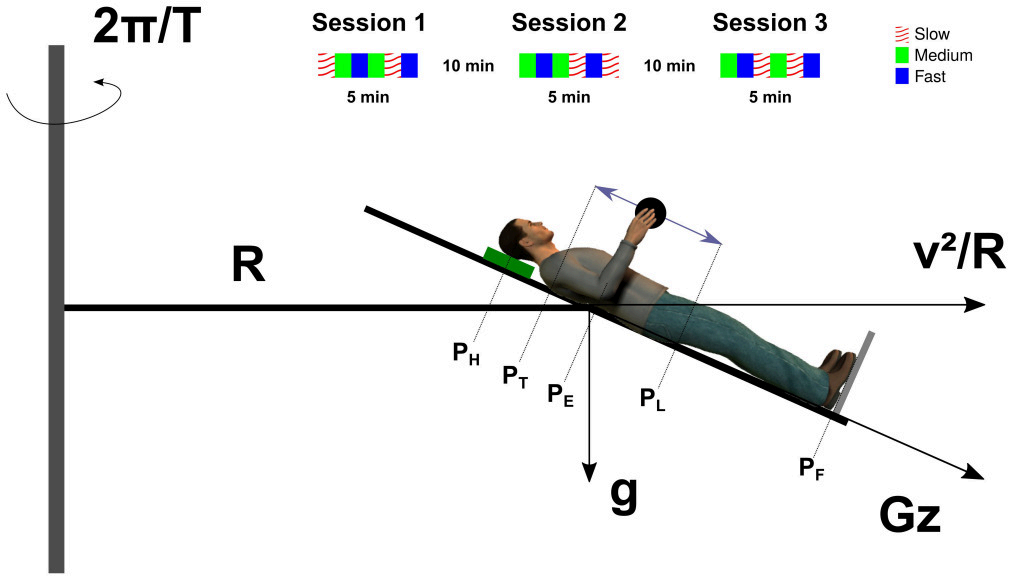
**Unscaled sketch of the participant in the SAHC**. The leftward gray vertical thin rectangle represents the axis of rotation about which the centrifuge rotates at an angular rate of $\frac{2\pi }{T}.$ The bed was tilted by −24 degrees and positioned such that the elbow joint (P_*E*_) was at distance R from the axis of rotation. The participant was supine on the bed, her/his head resting on a cushion (green rectangle) and the feet supported by a metallic plate (gray line). The vector Gz is the gravitoinertial resultant between the centripetal acceleration (horizontal vector) and the gravitational acceleration (vertical vector). The double arrow represents the trajectory of the object (black disk) in the sagittal plane. The upper inset illustrates a complete experiment composed by three sessions. Each color corresponds to a different pace condition (see legend). Symbols: P_H_, head; P_T_, top of trajectory; P_E_, elbow; P_L_, lower part of trajectory; and P_F_, feet.

Participants underwent three centrifugation sessions, each lasting 5 min (Figure 1). These sessions were separated by 10-min breaks during which participants rested supine and quietly while the centrifuge was idle. During centrifugation and following a signal from the operator, participants performed rhythmic upper arm movements in the sagittal plane with an instrumented object held in precision grip. The device recorded the 3-d forces and torques (mini40 force-torque sensor, 0.04 kg, ATI Industrial Automation, NC, USA). A 3 d accelerometer was also embedded in the object (TSD109C Tri-Axia, BIOPAC, ±5 g, 0.017 kg, CA, USA). All signals were continuously sampled at 200 Hz through a DAQ board (NI USB 6211, National Instruments, Austin, TX) and stored on a computer laptop strapped on the centrifuge. Participants were trained to produce trajectories parallel to the long (head-to-foot) body axis. Movement pace was provided by a metronome that emitted 2 auditory signals per cycle, one at the top and one at the bottom of the trajectory. The rhythm was controlled by the operator and routed via headphones to the subject's ears. Three paces (*Slow* = 0.7 Hz, *Medium* = 1 Hz and *Fast* = 1.3 Hz) were presented twice each for 30 s (3 paces × 2 repetitions × 30 s = 3 min). Pauses of about 20 s separated movement conditions in order to prevent fatigue and also to ensure that a good contact was maintained with the participant. Pace order was randomized and counterbalanced across participants. At the end of each session, the centrifuge went back to idle position and the subject was debriefed.

### Short arm human centrifuge configuration

Previous experiments extensively tested grip force adaptation to load force (LF) when either mass (*m*), acceleration (*a*) or gravity (*g*) were altered, separately or in combination (White et al., 2005; White, 2015), *LF*(*t*) = *mg* + *ma*(*t*). In this experiment, we set out to investigate how grip force is adjusted to load force when the gravitoinertial resultant also explicitly depended on position, *LF*(*x*,) = *mg*(*x*) + *ma*(*t*).

A short arm human centrifuge offered a unique opportunity to separate out the effects of time and space on the adaptation process of grip force to load force. Indeed, in contrast to a long radius human centrifuge, the resultant between centripetal acceleration induced by the rotation of the centrifuge and veridical gravity varies more for a given amplitude of movement close to the rotation axis than far from the rotation axis. In other words, gravitoinertial gradients are larger when approaching the center of rotation. Consider a point mass *m* situated at a horizontal distance *R* from the axis of rotation (Figure 1). This object is rotated at a constant angular velocity $\omega =\frac{2\pi }{T}$, with T being the period of rotation of the centrifuge, and is moving at a constant velocity $v=\frac{2\pi R}{T}$, tangent to the circular trajectory. This mass is subjected to both a constant gravitational acceleration directed downward (Figure 1, *g*) and to the centripetal acceleration (Figure 1, $a_{c}=\frac{v^{2}}{R}$). Therefore, when *m* is translated by a distance x along the radius, the magnitude of the gravitoinertial vector (Figure 1, *Gz*(*x*), in units of g) is given $Gz\left(x\right)=\frac{1}{g}\sqrt{\frac{16\pi^{4}{\left(R+x\right)}^{2}}{T^{4}}+g^{2}}$. We identified the centrifuge and geometrical parameters that maximized the gravitoinertial gradient. In other words, we adjusted *T*, movement space [*R, R* + *x*] and bed inclination angle such that $\frac{\partial Gz\left(x\right)}{\partial z}$ was maximal. We also had to take both ethical and technical constraints into account as some values of these parameters either could not be handled by the centrifuge or would have generated strong motion sickness. Details of this mathematical optimization process are presented in the Appendix of Supplementary Material. The centrifuge completed one revolution in 2.09s, the bed was tilted 24° downward and the elbow was positioned at 1.39 m from the axis of rotation. This configuration allowed us to induce a 0.4 g-gradient between both extremes of the hand trajectory (Figure 1, P_*T*_ and P_*L*_) which is a very strong perturbation and unnatural. The five positions (P_*H*_, P_*T*_, P_*E*_, P_*L*_, P_*F*_ in Figure 1) were subjected to different gravitoinertial vectors. Table 1 reports for each point, its horizontal distance from the axis of rotation, the magnitude of the centripetal acceleration and the magnitude and direction of the gravitoinertial vector.

**Table 1:** **Resultant dynamics at five points along the head-to-foot body axis (tilted 24° downward) placed in the centrifuge (one revolution in 2.09 s)**.

**Position**	**X distance (m)**	**|Centripetal Acc| (ms^−2^)**	**|Gz| (ms^−2^)**	**dir(Gz) (deg)**
P_H_	0.821	0.76	1.25	−52.8
P_T_	1.190	1.10	1.49	−42.3
P_E_	1.390	1.28	1.63	−37.9
P_L_	1.755	1.62	1.90	−31.7
P_F_	2.363	2.18	2.40	−24.6

*The elbow was positioned at 1.39 m from the axis of rotation. The first column denotes positions as illustrated in Figure 1 (P_H_, head; P_T_, top of trajectory; P_E_, elbow; P_L_, lower part of trajectory; and P_F_, feet). The next columns report, for each point: horizontal distance from the axis of rotation (X-distance), magnitude of centripetal acceleration (|Centripetal Acc|, horizontal vector in Figure 1), magnitude (|Gz|) and direction (dir(Gz) of the gravitoinertial resultant (oblique vector in Figure 1)*.

### Model of the task

In this section, we develop a simple model of the task that allows us to identify differences between acceleration signals when we take into account the effects of the centrifuge or not. Portions of cycles for which these differences are the largest are of particular interest. Indeed, we expect grip forces to be proportional to the real inertial variations.

Participants moved a small object (mass = 0.057 kg) in a non-inertial reference frame along a straight tilted trajectory in the sagittal plane. The accelerometer embedded in the instrument recorded the resultant vector of three accelerations: (1) the Earth constant gravitational attraction, (2) a centripetal acceleration due to the rotation of the centrifuge and (3) the acceleration induced by the movement of the object by the participant. Therefore, the load force that had to be counteracted is given by:

(1) $$\begin{array}{l} \vec{LF}=m\left(\vec{g}+{\vec{G}}_{z}\left(x\right)+{\vec{a}}_{m}\right) \end{array}$$

The first term is constant both in direction and magnitude. The second term varies in amplitude in function of the radial distance x from the axis of rotation. In this section, we quantify how the third term interacts with the two others and we model how pace affects the time course of the acceleration signal within a cycle, and for the three experimental paces.

Let us define a Cartesian reference frame centered on P_E_, with the x-axis and y-axis pointing rightward and upward, respectively. Rhythmic movements were performed on a straight line between P_L_ and P_T_, starting at the neutral position, i.e., between P_L_ and P_T_. The vectors (*x, y*), (ẋ, ẏ) and (ẍ, ÿ) denote position, velocity and acceleration, respectively. These trajectories are well described with sine waves, both for the x and y components:

(2) $$\begin{array}{l} \{\begin{array}{c} x\left(t\right)=x_{i}+\frac{1}{2}\left(x_{f}-x_{i}\right)\left(\text{sin }2\pi ft+\frac{1}{2}\right)\\ y\left(t\right)=y_{i}+\frac{1}{2}\left(y_{f}-y_{i}\right)\left(\text{sin }2\pi ft+\frac{1}{2}\right) \end{array} \end{array}$$

The parameters *x*_*i*_, *x*_*f*_, *y*_*i*_ and *y*_*f*_ are the initial (subscript *i*) and final (subscript *f*) positions in x and y and f is the frequency of movement. Velocity and acceleration are obtained by successive derivations of Equation (2):

(3) $$\begin{array}{l} \{\begin{array}{c} \dot{x}\left(t\right)=\pi f\left(x_{f}-x_{i}\right)\text{cos }2\pi ft\\ \dot{y}\left(t\right)=\pi f\left(y_{f}-y_{i}\right)\text{cos }2\pi ft \end{array} \end{array}$$

and

(4) $$\begin{array}{l} \{\begin{array}{c} \ddot{x}\left(t\right)=2\pi^{2}f^{2}\left(x_{i}-x_{f}\right)\text{sin }2\pi ft\\ \ddot{y}\left(t\right)=2\pi^{2}f^{2}\left(y_{i}-y_{f}\right)\text{sin }2\pi ft \end{array} \end{array}$$

One can now easily calculate the respective acceleration vectors involved in Eqaution (1):

(5) $$\begin{array}{l} \begin{array}{c} \vec{g}=\left(0,-g\right)\\ {\vec{G}}_{z}\left(x\right)=\left(\frac{4\pi^{2}\left(\text{R}+\text{x}\right)}{T^{2}},0\right)\\ {\vec{a}}_{m}=\left({\vec{a}}_{mx},{\vec{a}}_{my}\right) \end{array} \end{array}$$

**Figure 2:**
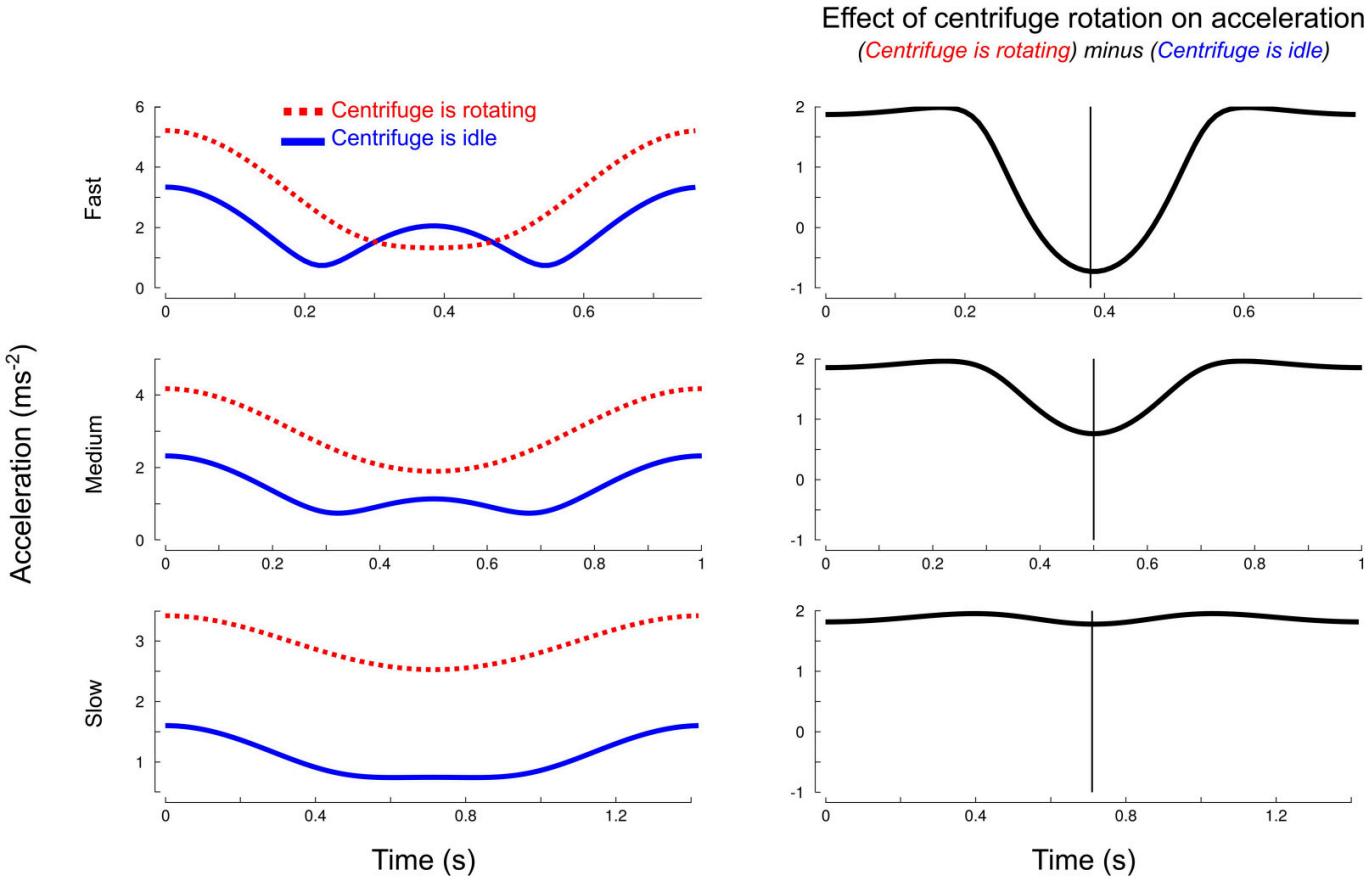
**Simulated effects of centrifuge rotation on the magnitude of total object acceleration over time**. Left column: total resultant acceleration ($\left||\vec{g}+\frac{{\vec{v}}^{2}}{R}+{\vec{a}}_{m}|\right|$, red dotted trace) and resultant acceleration without taking into account the centripetal acceleration ($\left||\vec{g}+{\vec{a}}_{m}|\right|$, blue solid trace) for each pace (three rows). Right column: magnification of the effects of centrifugation by subtracting $\left||\vec{g}+{\vec{a}}_{m}|\right|$ from $\left||\vec{g}+\frac{{\vec{v}}^{2}}{R}+{\vec{a}}_{m}|\right|$. The vertical cursor marks the largest discrepancies between the two accelerations.

The centripetal acceleration ${\vec{G}}_{z}$ and the acceleration generated by the participant depend on object position. Figure 2 (left column) depicts, for each pace (three rows) the resultant acceleration with ($\left||\vec{g}+{\vec{G}}_{z}\left(x\right)+{\vec{a}}_{m}|\right|$, red dotted trace) and without ($\left||\vec{g}+{\vec{a}}_{m}|\right|$, blue trace) taking into account the effects of the rotation of the centrifuge. It shows that there are differences between pace conditions but also within the time course of a single cycle. The largest differences, in proportion to the total amplitude of acceleration, are 52% for the fast pace, 28.8% for the medium pace and 5.1% for the slow pace. Interestingly, the contribution of ${\vec{G}}_{z}$ strongly depends on the phase of the cycle, especially for the two fastest paces. The subtraction between the traces depicted in Figure 2 (right column) magnifies how the rotation of the centrifuge contributes to total acceleration and, hence, load force. The largest differences occur at 76.6, 75.2, and 75.5% from cycle onset for fast, medium and slow paces, respectively (vertical cursors). These instants correspond to the lowest part of the trajectory.

### Data processing and analysis

Force and acceleration signals were smoothed with a zero phase-lag autoregressive filter (cutoff 10 Hz). A trial was defined as a series of cyclic movements. On average, per trial, participants performed 19.5 cycles for 0.7 Hz (SD = 6.9), 20.9 cycles for 1 Hz (SD = 2.2) and 26.3 cycles for 1.3 Hz (SD = 2.5). The largest number of cycles common to all conditions was 17. We analyzed trials and cycles separately. Furthermore, since load force varied differently within a cycle whether we take into account the effects of the rotation or not, we also analyzed four phases of the cycle.

Quantile-quantile plots were used to assess normality of the data. Repeated measures ANOVA was conducted on cycle frequency, grip forces and on the regression coefficients between grip force and load force. When relevant, we assessed the effects of *Session* (1, 2, or 3), *Frequency* (0.7, 1, or 1.3 Hz), *Repetition* (1 or 2), *Cycle* (1 to n) and *Phase* (1, 2, 3, or 4) on the above variables. Post-hoc comparisons were made using Fischer least significant differences (LSD). Paired *t*-tests of individual subject means were used to investigate differences between conditions on the above variables. Data processing and statistical analyses were done using Matlab (The Mathworks, Chicago, IL). We report partial eta-squared values for significant results (*p* < 0.05) to provide indication on effect sizes.

## Results

Participants cyclically moved an instrumented object along the long body axis aligned with the gravitoinertial direction during rotation in a human centrifuge. Here, we challenged the limits of the adaptation capacity of the motor system by assessing how participants controlled grip force when load force comprised a gravitoinertial component that varied explicitly with local vertical position. The generation of such dynamics can only be tested in a short arm human centrifuge.

We verified that participants adopted a pace that matched the instructions. We used a Fast Fourier Transform to extract the main frequency component of the acceleration profile for each trial. A 2-way ANOVA confirmed a main effect of *Frequency* [*F*_(2,139)_ = 143.5, *p* < 0.001, $\eta_{p}^{2}=0.66$] and *Session* [*F*_(2,139)_ = 3.4, *p* = 0.038, $\eta_{p}^{2}=0.02$] on real movement frequency. Paired *t*-tests revealed no difference between actual and theoretical rhythms for 1 and 1.3 Hz [both *t*_(18)_ = 1.8, *p* = 0.084], but faster paces for the slowest condition [0.79 vs. 0.7 Hz, *t*_(18)_ = 3.1, *p* = 0.006, $\eta_{p}^{2}=0.35$].

Frequency, acceleration and load forces are linked through Equations (1) and (4). A 3-way ANOVA (factors: *Frequency, Session*, and *Cycles*) revealed higher peaks of acceleration in high frequency conditions [*F*_(2, 2,276)_ = 144.1, *p* < 0.001, $\eta_{p}^{2}=0.11$ $\eta_{p}^{2}=0.11$], which also induced larger peak load forces [*F*_(2, 2,276)_ = 144.1, *p* < 0.001, $\eta_{p}^{2}$ = 0.11 $\eta_{p}^{2}$ = 0.11]. As reported previously (Flanagan and Wing, 1995, 1997), participants adopted grip forces proportional to peak load forces, as revealed by proportional peak grip forces [*F*_(2, 2,276)_ = 15, *p* < 0.001, $\eta_{p}^{2}$ = 0.01$\eta_{p}^{2}$ = 0.01].

A first question arises as to how the tight link between grip and load forces was affected by *Frequency* and whether it was influenced by time. To quantify this relationship, we calculated, for each cycle of movement, the best linear fit between these two time series (Flanagan and Wing, 1995; Hejdukova et al., 2002; Zatsiorsky et al., 2005). Participants accomplished the task for three sessions (*Session*), each frequency was repeated twice per session (*Repetition*) and each repetition involved at least 17 cycles (*Cycles*). We could therefore analyze adaptation at three different time scales. The 4-way ANOVA revealed significant increases of gains [*F*_(2, 3,606)_ = 31.1, *p* < 0.001, $\eta_{p}^{2}=0.02]$ and offsets [*F*_(2, 3,606)_ = 7.9, *p* < 0.001, $\eta_{p}^{2}<0.01$] with *Frequency*. Furthermore, gains significantly increased across *Session* [*F*_(2, 3,606)_ = 4.6, *p* = 0.01, $\eta_{p}^{2}<0.01$] and *Repetition* [*F*_(1, 3,606)_ = 10.6, *p* = 0.001, $\eta_{p}^{2}<0.01$]. In contrast, offset significantly decreased across *Session* [*F*_(2, 3,606)_ = 22.4, *p* < 0.001, $\eta_{p}^{2}=0.01$] and *Repetition* [*F*_(1, 3,606)_ = 66, *p* < 0.001, $\eta_{p}^{2}=0.02$]. However, we did not observe any effect of *Cycle* on these two parameters [gains: *F*_(16, 3,606)_ = 0.8, *p* = 0.714; offset: *F*_(16, 3,606)_ = 0.7, *p* = 0.761]. To sum up, while *Frequency* induced larger slopes and safety margins, participants tended to optimize the task by simultaneously increasing the gain and lowering grip force. This adaptation occurred within a trial but not between trials.

**Figure 3:**
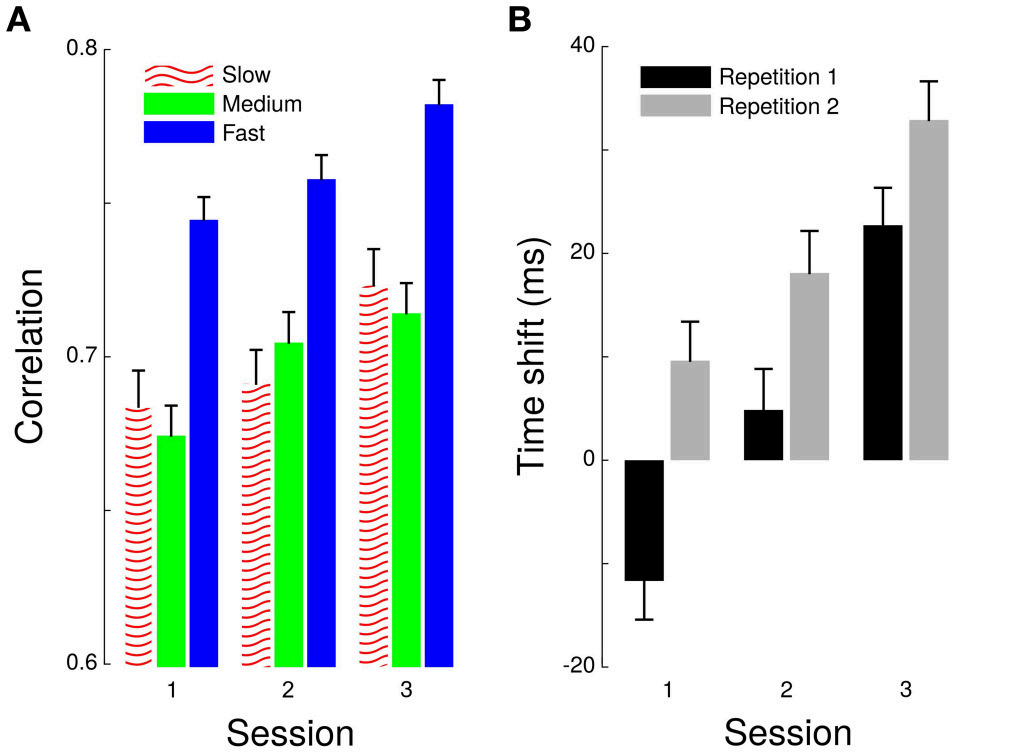
**Cross-correlation between grip and load forces**. Largest coefficient of correlation between grip and load forces **(A)** and the time shift for which this condition was fulfilled **(B)**. Correlations are shown across *Sessions* (x-axis) and separately for each *Frequency* (see legend). Time-shifts are also depicted across *Sessions* (x-axis) but separately for Repetition 1 (black bar) and Repetition 2 (gray bar) of frequency.

Parameters of a linear regression do not provide indications on goodness of fit. Therefore, we pushed our analyses one step further by considering the cross-correlation between grip and load forces within each cycle. This procedure provided an estimate of the overall synergy between the two forces. Correlations quantified how well grip and load force profiles matched, which indicated the accuracy of scaling of grip force. Time-shifts provided a measure of the asynchrony between the two forces. A positive time-shift signaled an anticipatory grip force. A 4-way ANOVA reported significant effects of *Frequency* [*F*_(2, 2,759)_ = 42.4, *p* < 0.001, $\eta_{p}^{2}=0.03$] and *Session* [*F*_(2, 2,759)_ = 12.7, *p* < 0.001, $\eta_{p}^{2}=0.01$] on this best correlation coefficient (Figure 3A). A *post hoc t*-test revealed that fast pace induced better correlations than slow [*t*_(17)_ = 4.3, *p* = 0.001 $\eta_{p}^{2}=0.52$] and medium paces [*t*_(17)_ = 5.6, *p* < 0.001, $\eta_{p}^{2}=0.65$] and that the two slower paces were not different [*t*_(18)_ = 1.4, *p* = 0.189]. Furthermore, the time-shift (Figure 3B) increased across *Session* [*F*_(2, 2,759)_ = 17.2, *p* < 0.001, $\eta_{p}^{2}<0.01$] and from *Repetition 1* to *Repetition 2* [*F*_(1, 2,759)_ = 11.6, *p* < 0.001, $\eta_{p}^{2}<0.01$] but not across *Frequency* [*F*_(2, 2,759)_ = 0.2, *p* = 0.807]. Therefore, the synergy between grip force and load force improved across *Session*, participants adopting a more predictive behavior underlined by increasing time-shifts.

**Figure 4:**
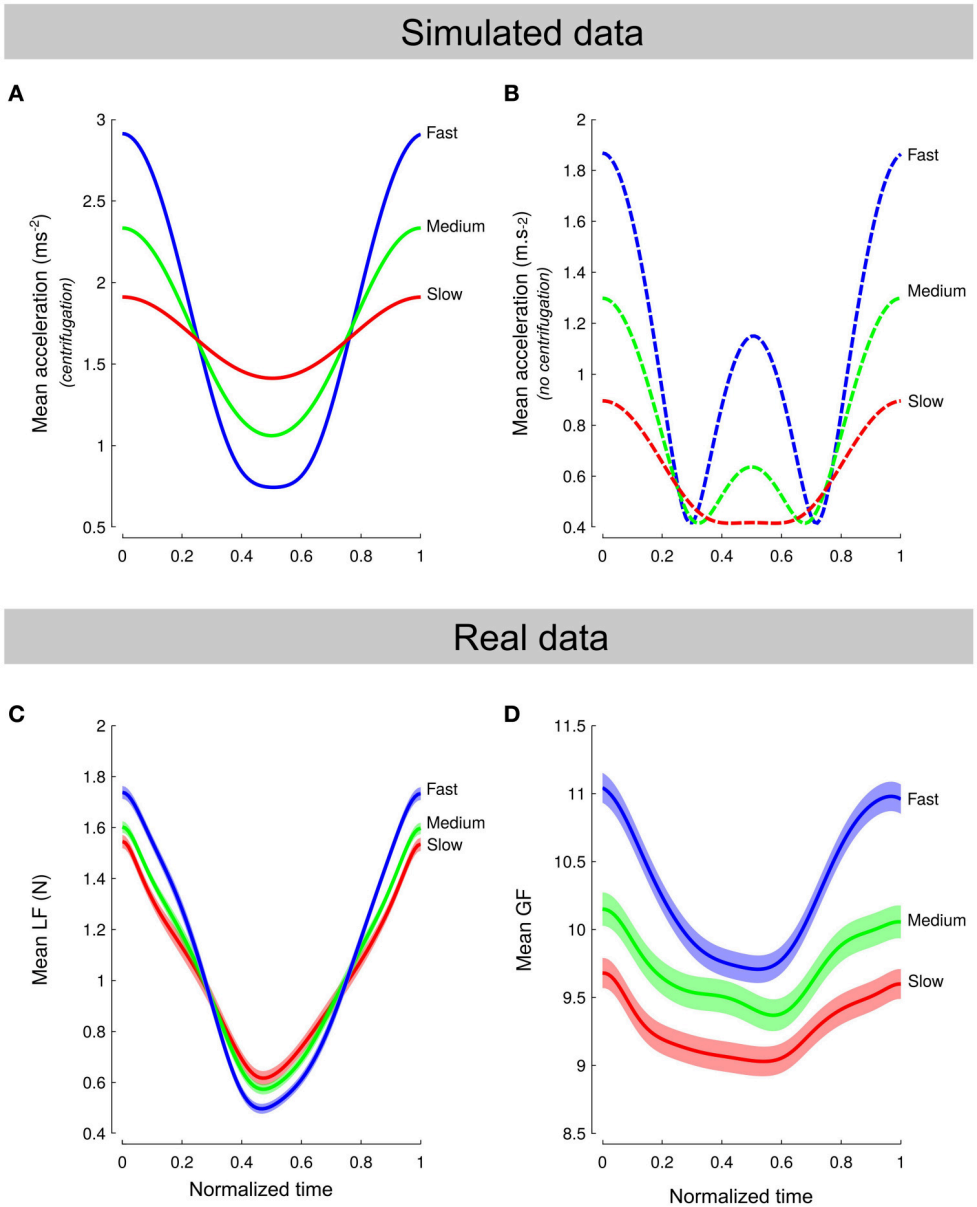
**Comparison between model and data. (A,B)** Simulated load force over normalized time when the model takes into account the effect of the rotation on the object **(A)** or not **(B)**. Colored lines correspond to a different frequency. **(C,D)** Actual averaged load force cycles **(C)** and grip force cycles **(D)** normalized across all conditions. Note that the pattern of load forces in **(C)** span a shorter force amplitude than simulated accelerations in **(A)** because the object mass was small.

Centrifugation added a position-dependent acceleration component that contributed to the total inertial force, resulting in an unusual perturbation. Figure 4A depicts simulated load force over normalized time when the model takes into account the three sources of accelerations (i.e., constant gravity, cyclic movement and centripetal accelerations). It shows that the amplitude of the signal was proportional to frequency. Figure 4B also shows simulated data but without taking into account the effects of the rotation. The model predicts very different patterns of acceleration and, hence, load force, if we include or not the effects of the centrifugation. Actual load force traces (Figure 4C, averaged normalized cycles across all conditions) clearly resemble the model that includes all acceleration terms (Figure 4A). In particular, the three amplitudes were significantly different between *Frequency* [*F*_(2, 139)_ = 15.8, *p* < 0.001, $\eta_{p}^{2}=0.18$] while the average load forces were similar [*F*_(2, 139)_ = 0.1, *p* = 0.872]. Furthermore, modeled load force traces intersected at 25 and 75% from cycle onset which is very close to what we observed in real data (28.6 and 74.5%).

**Figure 5:**
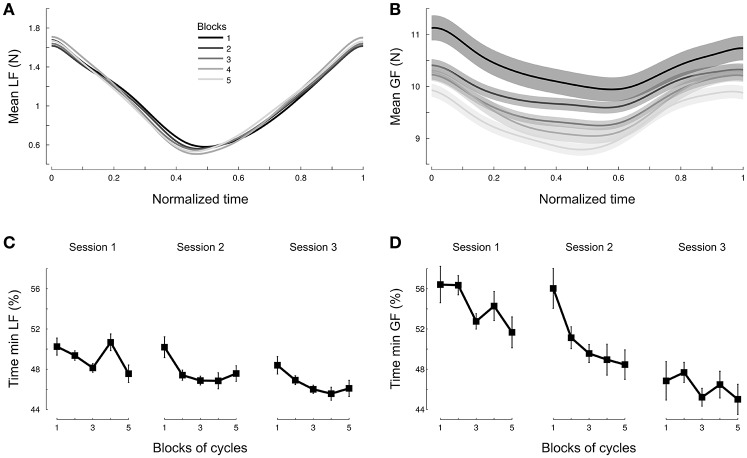
**Participants adjust grip force but not load force across cycles**. Averaged load force profile during one cycle **(A)** and averaged grip force profile during one cycle **(B)** normalized across all conditions and depicted separately for each block of continuous cycles. Blocks 1 to 4 pool 3 cycles together and block 5 includes the last five cycles. The earlier blocks in the trial are depicted in dark gray and late blocks are shown in light gray [see legend in **(A)**]. The occurrence of minimum load force **(C)** and grip force **(D)** within a cycle is plotted as a function of block. The three sessions are shown separately. Time is normalized by cycle length in all panels.

Participants should have anticipated the actual load force profile by adjusting grip force. Data show that participants exerted grip forces that paralleled the actual load forces and not the one they might have predicted without taking into account the effects of the centripetal acceleration (Figure 4D). A natural question arises as to whether the behavior observed in Figure 4D was reached immediately upon exposure to the environment or needed time to settle. To quantify this, we formed five blocks of continuous cycles and plotted averaged force traces across blocks. Figure 5 depicts these five averaged traces for load force (Figure 5A) and grip force (Figure 5B). It shows first that load force traces overlap well (Figure 5A). In contrast, grip forces exhibit a continuous progression between early (Figure 5B, dark lines) and late grip force traces (Figure 5B, light lines). While amplitudes gradually decreased across *Blocks* [*F*_(4, 684)_ = 15.8, *p* < 0.001, $\eta_{p}^{2}=0.04$], the occurrences of minimal grip forces shifted sooner in the cycle.

To deepen these analyses, we focused on the normalized time at which the minimal forces were reached. We found that minimal load forces occurred on average 48.2% after cycle onset (Figure 5C) and did not vary between *Session* [*F*_(2, 263)_ = 2.4, *p* = 0.09], *Repetition* [*F*_(1, 263)_ = 2.7, *p* = 0.103] or *Blocks* [*F*_(4, 263)_ = 2.2, *p* = 0.119]. In contrast, the same analysis conducted on grip forces reported an initial skewness of 57% in grip force profiles (Figure 5D, Session 1, Blocks 1-2) that gradually decreased with *Session*. We confirmed this observation statistically. The 3-way ANOVA reported a main effect of *Session* [*F*_(2, 263)_ = 8.6, *p* < 0.001, $\eta_{p}^{2}=0.04$], *Repetition* [*F*_(1, 263)_ = 29.4, *p* < 0.001 $,\;\eta_{p}^{2}=0.07$] and *Blocks* [*F*_(1, 263)_ = 4.4, *p* < 0.005, $\eta_{p}^{2}=0.03$] on this minimum grip force. Altogether, this demonstrates that a subtle modification of grip force occurs over time to match the actual and novel perturbation.

## Discussion

The purpose of this study was to test whether the successful adaptation usually reported in altered gravitoinertial environments is a consequence of the ability to predict the time course of the perturbation or results from a more complex process. Put differently, we tested participants' ability to switch and adapt to a gravitoinertial field induced by a short-arm centrifuge that explicitly varied with position. Apart from following the prescribed rhythmic tone, there were no further accuracy requirements. We addressed these questions by using the well-established grip force/load force coupling paradigm.

Motor adaptation to different dynamical contexts has been widely documented (Wolpert et al., 2011; Wolpert and Flanagan, 2016). To probe motor learning, scientists use robot-based paradigms to perturb a task with fixed and repeatable structures. For example, in a seminal study, Shadmehr and Mussa-Ivaldi used a robot to apply mechanical forces to the hand which revealed powerful error-based learning in the motor system (Shadmehr and Mussa-Ivaldi, 1994). In all investigations, the dynamics produced by the robot had a clear dependency on some movement parameters, such as the speed of the subject's hand. Furthermore, in most experiments, only the end effector or the upper limb is perturbed by the robot. Importantly, in these cases, the sensory system remains unaffected. When a motor error occurs, it is most likely attributed to the effector that sensed the perturbation by an unusual or uncontrollable phenomenon (White and Diedrichsen, 2010).

Parabolic flights, rotating-rooms and underwater settings allow circumventing these limitations as they coherently immerse participants in a new dynamical context. Adaptation of motor responses has been reported following changes in gravity during parabolic flights (Hermsdörfer et al., 1999; Augurelle et al., 2003; Mierau et al., 2008; Crevecoeur et al., 2009a), in gravitoinertial environments (Dizio and Lackner, 1995; Nowak et al., 2004; Göbel et al., 2006) and underwater (Macaluso et al., 2016). However, exposures were either constant or occurred in a reproducible manner and could eventually be predicted.

In the present experiment, we report a successful motor adaptation of grip force with load force in yet another context. A change of frequency induced larger accelerations and hence load forces. Participants followed the instruction generally well and could move the object at the correct frequency. Previous reports demonstrated the versatility of the motor system to match load forces even when movement pace is higher than 1 Hz (Flanagan et al., 1993; Zatsiorsky et al., 2005) or when load force frequency is multiplied by a factor 2 in weightlessness (Nowak et al., 2000; Augurelle et al., 2003). They were, however, slightly faster for the slowest pace during early exposure as shown previously (Augurelle et al., 2003; White et al., 2008). Further, the nature of the linear regression between load force and grip force changed with frequency as revealed by larger gains and offsets and better correlation coefficients. Offsets reflect the net grip force predicted by the linear model when load force is zero and can therefore be interpreted as a safety margin (Johansson and Westling, 1984, 1988; Cole and Johansson, 1993). Consistent with our results, previous work reported that gains decreased and offsets increased with movement frequency (Zatsiorsky et al., 2005; White, 2015). We found values of correlation coefficients compatible with other experiments (Flanagan et al., 1993). Finally, time-shifts that quantify feed forward processes were not affected by frequency.

Our paradigm allowed breaking down the experiment into different time scales. Our study used three sessions separated by 10-min pauses. Each pace was presented randomly twice per session and each trial was composed of a series of 10–20 cycles of movements. Increased gains, decreased offsets, improved correlation and more positive time-shifts between load and grip forces, all revealed that learning occurred over sessions and repetitions but not over contiguous cycles of movement. Despite the very stressful environment—5 participants (20%) became motion sick and could not complete the experiment—, grip to load force coordination improved over time. Noteworthy, grip forces were unnecessarily large (10–11 N) considering the light object mass. The presence of disease (Hermsdorfer et al., 2003), high complexity (Krishnan and Jaric, 2010), variability (Hadjiosif and Smith, 2015), or fatigue (Emge et al., 2013) usually translate in a deterioration of the above parameters.

Sessions were separated by idle time and repetitions were randomly interleaved. Participants performed context switches between conditions. Blocks, instead, were a succession of cycles within the same dynamical context. Interestingly, we did not observe forgetting between switches, which indicates participant's abilities to adjust their control early in the trial. In contrast, the capacity of the central nervous system to learn different task dynamics in different contexts has been proved to be limited (Gandolfo et al., 1996; Karniel and Mussa-Ivaldi, 2002) even when the change of direction of a perturbation is made fully predictable through the use of an alternating sequence (Conditt et al., 1997) or a predictive visual cue (Osu et al., 2004). Our data show that the central nervous system is capable of switching between different dynamics even when they contain highly unfamiliar components, such as a position-dependent gravitoinertial term. This adds to the list of previously observed experimental contexts in which switching is made possible (Cothros et al., 2006; Nozaki et al., 2006; White and Diedrichsen, 2013). One fundamental difference between our experimental context and those using robotic approaches and rotating chairs is the fact that the participants are completely immersed into a new environment. Indeed, healthy participants tested in robotic studies are endowed with somatosensory signals from the reaching arm while the rest of the body is not affected by the new dynamics. In contrast, some centrifuge investigations placed the subject's head aligned with the vertical axis of rotation, therefore preventing information from the vestibular system to contribute to motor adjustments (DiZio and Lackner, 2001; Nowak et al., 2004). It was indeed shown that deviations of the hand remain uncorrected when the patient's head is fixed in space during trunk rotations. However, adaptation occurred when the head moved with the trunk (Guillaud et al., 2011). It was proposed that vestibular signals may influence all stages of the sensorimotor pathway from a desired movement goal down to specific motor-unit innervation (Bockisch and Haslwanter, 2007). Neuroimaging protocols using small amplitudes of movements (Rousseau et al., 2016a), visual gravitational cues (Indovina et al., 2005) or resting states analyses in astronauts (Demertzi et al., 2016) reported the critical role of a vestibular network that may process gravity-relevant information in action planning and execution. However, different this novel dynamic is, we posit the switching is also made possible because low level multisensory signals are coherently affected which allows adaptation. We speculate the same phenomenon occurs during parabolic flights, when participants are exposed to a series of gravitational environments or underwater, when neutral buoyancy is exerted on body segments as opposed to body center of mass (Macaluso et al., 2016).

While learning a new task in different gravitational fields is surprisingly fast, sometime is necessary for the motor system to adjust subtle parameters underlying the action. One such parameter is the bias induced by gravitational and visual verticality. In reaching hand movements, the arm spends proportionately less of the total time to accelerate upward compared with downward and horizontal movements (Papaxanthis et al., 1998; Gaveau et al., 2016). It is now accepted that in order to save muscular effort, the brain integrates the assistive role of gravity to slow an upward movement and to accelerate a downward movement (Papaxanthis et al., 2003; Rousseau et al., 2016b). This translates into directional kinematic asymmetries. The same bias is responsible for the persistence of larger grip forces when moving an object upward compared to downward in weightlessness (White et al., 2012).

Here, the switching we observed was not incomplete. Whereas participants could produce stereotyped trajectories from the outset (Figure 5A), one subtle feature in the grip force profile needed time to settle (Figure 5B). Indeed, grip force cycles were asymmetric, exhibiting a minimum later in the movement cycle. In other words, participants produced a movement that was only efficiently mastered at the end of the experiment. This time parameter gradually adjusted across sessions and repetitions, with a forgetting only observable between the last block of Session 1 and the first block of Session 2. This behavior contrasts with the fact that people can learn to predict the consequences of their actions before they can learn to control them (Flanagan et al., 2003). We speculate that it is not the case here because the state of the sensorimotor system itself is altered by the environment. Although coherent, flows of sensory information are new and more time is necessary to accomplish fine adjustments.

To sum up, we have shown that the motor system can switch between different dynamical contexts never experienced before and that this is not a mere consequence of the ability to predict the time course of this new dynamics. Our results further confirm that the brain integrates the effects of the gravitoinertial environment to perform optimal actions and does not consider these effects as disturbances. Furthermore, our findings show that consistent sensory information born in a homogeneous context and from all sensory organs convey signals that can be efficiently processed by the brain to define a control policy and execute an action. We speculate that learning of new challenging motor tasks could be sped up by providing coherent multimodal sensory feedback, which has consequences when designing efficient rehabilitation protocols. Indeed, one recommendation for neuro-rehabilitation would be to provide to the patient multiple sensory inflows in parallel (e.g., vision, touch and audition) and not only one at a time. A straightforward prediction is that providing irrelevant multimodal sensory information should, instead, negatively impact learning and rehabilitation.

## Author contributions

OW: designed the experiment; MB, CR, and OW: recorded the data using the human centrifuge; MB: analyzed the data; MB and OW, wrote the manuscript; CP, provided feedback on the manuscript.

### Conflict of interest statement

The authors declare that the research was conducted in the absence of any commercial or financial relationships that could be construed as a potential conflict of interest.
