# Supplementary material for: Coherent Multimodal Sensory Information Allows Switching between Gravitoinertial Contexts
Source: Front Physiol. 2017 May 11;8:290. doi: 10.3389/fphys.2017.00290 (PMC5425486; doi:10.3389/fphys.2017.00290)

**Supplementary material**

A short arm human centrifuge allows to vary the resultant of the gravitoinertial vector significantly over a certain distance. In this appendix, we derive the centrifuge and geometrical parameters that maximize the gravitoinertial gradient $\frac{\partial Gz(x)}{\partial z}$, for Gz defined as:

|  | $Gz\left( x \right)=\frac{1}{g}\sqrt{\frac{16\pi^{4}{(R+x)}^{2}}{T^{4}}+g^{2}}$ | (A1) |
| --- | --- | --- |

We seek to find how much we have to move radially with respect to a certain fixed radius *R*, a period of rotation *T* and a gain $\gamma$, in order to have a gradient of $\gamma g$:

|  | $\left\vert Gz\left( R+x \right) \right\vert-\left\vert Gz\left( R \right) \right\vert=\gamma g$ | (A2) |
| --- | --- | --- |

where $\gamma=0.5$.

Figure A1 depicts iso-gravitational curves (left panel) in function of radius (x-axis) and period (y-axis). It shows that at a fixed period, the more R increases, the more Gz increases and, conversely, for a constant R, the shorter the period, the larger the Gz. Iso-gravitational curves are not parallel which underlines a nonlinear effect between R and T. The left panel of Figure A1 illustrates these combined effects in three dimensions.


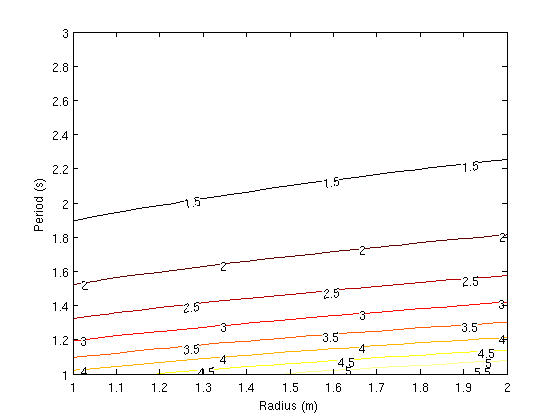

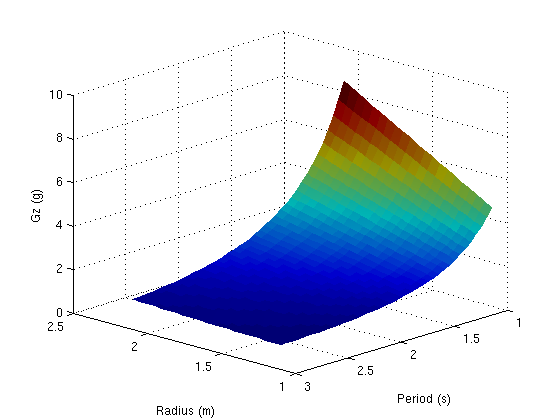


Plugging (1) into (2) and setting $\alpha^{2}≝\frac{16\pi^{4}}{T^{4}}$ yields to the following expression:

|  | $\sqrt{\alpha^{2}\left( R+x \right)^{2}+g^{2}}-\sqrt{\alpha^{2}R^{2}+g^{2}}=\gamma g$ | (A3) |
| --- | --- | --- |

Equation (3) can be rearranged in a form of a second order polynomial:

|  | $\alpha^{2}x^{2}+2\alpha^{2}Rx-\gamma^{2}g^{2}-2\gamma g\sqrt{\alpha^{2}R^{2}+g^{2}}=0$ | (A4) |
| --- | --- | --- |

By setting:

$${a=\alpha}^{2}$$

$$b=2\alpha^{2}R$$

$$c={-\gamma}^{2}g^{2}-2\gamma g\sqrt{\alpha^{2}R^{2}+g^{2}}$$

We can easily find the positive root of Eq. 4:

|  | $x=\frac{-b+\sqrt{b^{2}-4ac}}{2a}=f\left( T,R \right)$ | (A5) |
| --- | --- | --- |

The set of solutions is given in function of T and R and is illustrated in Fig. A2 (left panel) for periods and radii ranging from 1.84 s to 2.34 s and from 1.34 m to 1.44 m, respectively. Each colored line corresponds to a different combination of T and R that gives the same amplitude of movement (x in Eqs. 3-5) and that lead to a change of 0.5g over the horizontal component of the movement path. The radius of the centrifuge can mechanically be adjusted between 1.33 m and 1.74 m.


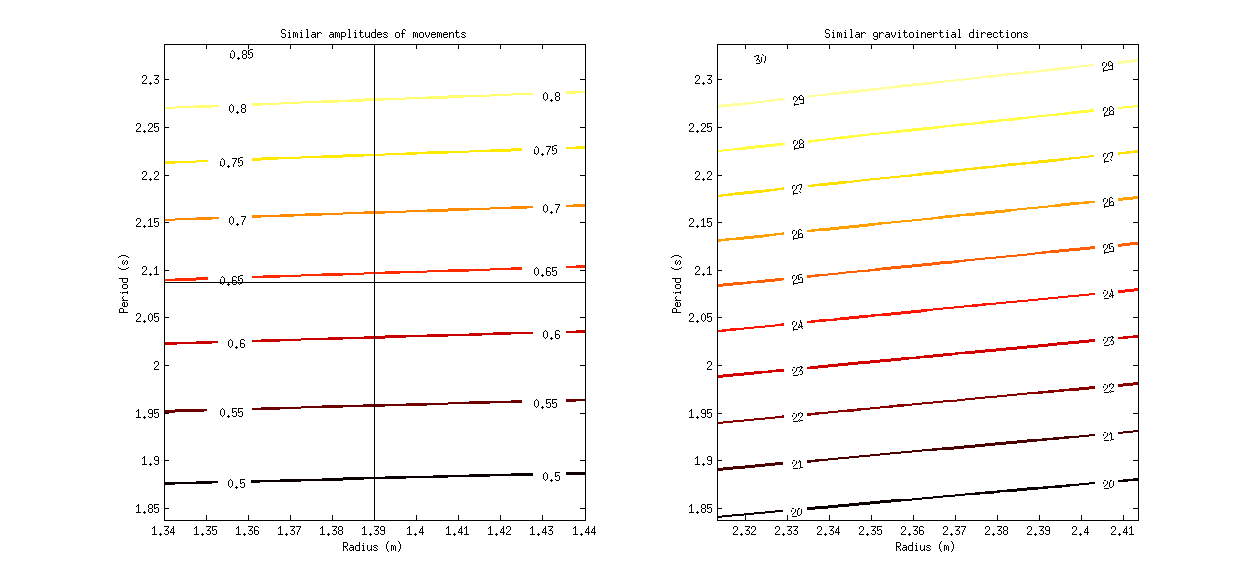


We also had to match the angular inclination of the bed with the direction of the gravitoinertial vector at the feet for ethical and scientific reasons. On the one hand, this optimized postural stability by allowing participants to have a strong contact with the metallic plate. On the other hand, the plates provided a reactive push to the body and reinforced the impression of increased weight through feet mechanoreceptors. Furthermore, movement performed in a rotating – hence non inertial – reference frame -, contribute to Coriolis effects, proportional to velocity components not parallel to the axis of rotation. Therefore, we also had to keep T reasonably low. By referring to anthropomorphic tables, we estimated the length between elbow and the feet to be 0.97 m. (Winter, 2009). The direction (in deg) of the gravitoinertial vector is given by:

|  | $\beta={tan}^{-1} \left( \frac{gT^{2}}{4\pi^{2}R} \right)$ | (A6) |
| --- | --- | --- |

Figure A2 (right panel) depicts the combinations of T and R that correspond to similar angles. The centrifuge bed could be tilted between 0 deg (horizontal) and -51 deg.

Taking all these constraints into account, a reasonable compromise was found for an angle of -24.62 degrees, which lead to a magnitude of the gravitoinertial resultant at the feet to 2.4g, which is still acceptable for the protocol (<2.5g at the feet). If we position the closest point of the object trajectory along the radius axis at 1.39 m (Fig. A2, left panel, vertical cursor) and if the centrifuge rotates with a period of 2.05 s (Fig. A2, left panel, horizontal cursor), the range of movement that allows to undergo a 0.5-g change over its amplitude is $x=0.64 m$. In other words, the movement interval is $\left[ R;R+y \right]=\left[ 1.39 m;2.03 m \right]$. For an inclination angle of -24 deg, this corresponds to movement amplitudes *d* along the bed axis:

|  | $d=\frac{x}{\cos\left( 24 \right)}=0.70m$ | (A7) |
| --- | --- | --- |

In conclusion, subjects were positioned such that their elbow contacted the bed at x=1.39 m from the axis of rotation. The neutral position of the elbow was such that the forearm was perpendicular to the bed at the elbow joint (see Fig. 1). The plate at the feet was adjusted to each participant’s height. Note that variability in height was small (maximal admissible height of 1.8m and see Methods). Trajectories were performed about the elbow over an angular amplitude of 135 deg, resulting from a forward extension of 90deg and a backward flexion of 45deg. With mean elbow-wrist lengths of *e* (0.37 m, REF), this leads to $e\left( 1+sin45 \right)$ along the bed. Figure A3 plots the direction (left axis) and magnitude (right axis) of the gravitoinertial vector in function of R covering the x-space identified for this experiment. First, it shows that the direction varies over an amplitude of 15 degrees which is relatively small and mostly aligned with bed inclination and the felt vertical orientation. Second, the difference between maximal and minimal magnitude of the gravitoinertial vector is 0.49g, which is very close to what we targeted (0.5g).


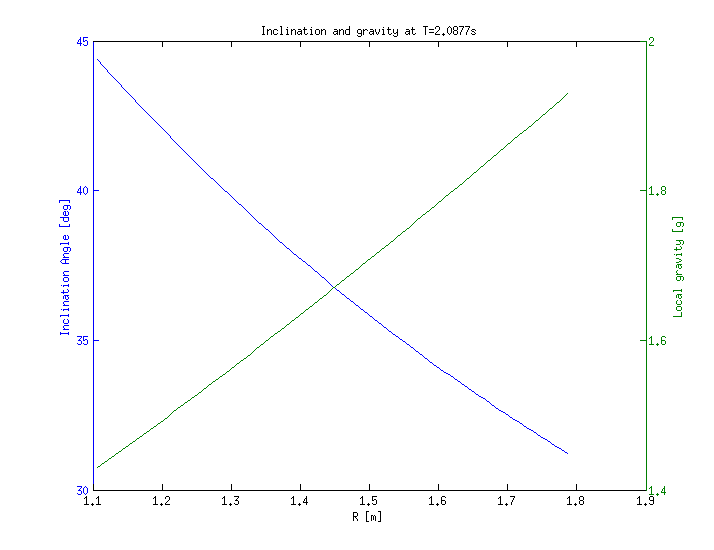

Supplement: Supplementary file 1 [file DataSheet1.DOCX]
